# The relationship between gender, educational background, and learning environment with nursing student’s anxiety

**DOI:** 10.1590/0034-7167-2022-0615

**Published:** 2024-12-16

**Authors:** Amelia Margaretha Simbolon, Jesika Sari Munthe, Mikar Syah Idaman Daeli, Ineke Patrisia, Chriska Roully Adeline Sinaga

**Affiliations:** I Universitas Pelita Harapan. Tangerang, Banten, Indonesia

**Keywords:** Anxiety, Education, Learning, Nursing, Students., Ansiedade, Educação, Aprendizagem, Enfermagem, Estudantes., Ansiedad, Educación, Aprendizaje, Enfermería, Estudiantes.

## Abstract

**Objectives:**

to identify variables associated with nursing students’ anxiety-related clinical practice.

**Methods:**

this study used a correlational quantitative descriptive design with the STROBE equator instrument. The population is 233 nursing students with a sample of 135. Data were collected from March to April 2022 using validated instruments.

**Results:**

the Chi-square test and the sig likelihood ratio on the gender, educational background, and learning environment is more than 0.05 so there is no significant relationship between the variables and student anxiety.

**Conclusions:**

students need to be prepared again before entering the practice field. Qualitative research is needed too.

## RESUMO

**Objetivos:**

identificar as variáveis associadas à prática clínica relacionada à ansiedade dos estudantes de enfermagem.

**Métodos:**

foi utilizado um delineamento descritivo, quantitativo e correlacional com as diretrizes do STROBE, EQUATOR. A população foi de 233 estudantes de enfermagem com uma amostra de 135 indivíduos. Os dados foram coletados entre março e abril de 2022 com o uso de instrumentos validados.

**Resultados:**

o teste do qui-quadrado e a razão de verossimilhança significativa para sexo, formação educacional e ambiente de aprendizagem são maiores que 0,05, logo, não há relação significativa entre as variáveis e a ansiedade do estudante.

**Conclusões:**

os estudantes precisam ser preparados novamente antes da entrada no campo de prática. Pesquisas qualitativas também são necessárias.

## RESUMEN

**Objetivos:**

identificar las variables asociadas a la práctica clínica relacionadas con la ansiedad de los estudiantes de enfermería.

**Métodos:**

se utilizó un diseño descriptivo, cuantitativo, correlacional con las recomendaciones STROBE, EQUATOR. La población fue de 233 estudiantes de enfermería con una muestra de 135 personas. Los datos se recopilaron de marzo a abril de 2022 utilizando instrumentos validados.

**Resultados:**

la prueba de chi-cuadrado y razón de verosimilitud significativa para género, formación académica y entorno de aprendizaje son superiores a 0.05, por lo que no existe una relación significativa entre las variables y la ansiedad de los estudiantes.

**Conclusiones:**

los estudiantes necesitan prepararse nuevamente antes de ingresar al campo de la práctica. Investigaciones cualitativas también son necesarias.

## INTRODUCTION

Education and learning for all people at all levels is an important agenda for sustainable human development. This is implied in Sustainable Development Goal 4 which states “*Ensure inclusive and equitable quality education and promote life-long learning opportunities for all*”^(1)^. Learning is essential for conveying knowledge using various methods that support students to learn optimally. Lestari^(2)^ states that the learning process in nursing is a necessary process that nursing students must pass to achieve broad knowledge by paying attention to ethical and moral principles in preparing students to become professional nurses. The learning process in Indonesia is carried out by delivering theoretical and practical learning materials to improve skills^(3)^.

Theoretical and practical learning is essential to achieve specific learning objectives for nursing students^(4)^. Nursing students are expected to balance theory and practice, which they can apply through hospital clinical practice. Clinical practice is an independent action by nursing students to provide nursing care according to the intervention^(5)^. According to Sari^(6)^, nursing students prepare physically and psychologically during clinical practice. One of the psychological aspects experienced by nursing students during clinical practice is anxiety. Anxiety is the emergence of a person’s emotions when experiencing problems indicated by worry, which can have a physical effect^(7)^.

This study aims to identify the relationship of the variables of nursing students’ anxiety related to clinical practice. A preliminary study was conducted on November 19, 2021, on 25 2^nd^ year nursing students and found that 25% of students experience anxiety because they are not ready to participate in clinical practice, 30 % are worried about their knowledge, and 15% are anxious because they did not know the clinical practice environment, and 30% were anxious because they lacked experience attending clinical practice. The reason is, during the Covid-19 pandemic, clinical skills learning which was usually carried out face-to-face in a laboratory/ practice field with assistance from a lecturer/clinical educator, had to be carried out online. According to Stuart *et al.* ^(8)^, the higher the anxiety, the less perceptual and focused a person will be. This claim is supported by Kusumastuti^(9)^, who claims that there is a relationship between student anxiety and academic achievement.

Based on the research conducted by Vellyana et al.^(10)^ states that there is a relationship between gender with anxiety, with a chi-square test result of 0.043. This study shows that 90.4 of % female students experience related anxiety in clinical practice. The results of this study align with the research by Padila in Sugiharno et al.^(11)^, explain that the gender of the respondents who experienced the most anxiety is a woman with a mild level of anxiety. Research by Kaplan & Saddock in Jayanti, Krisnawati & Devi^(12)^ stated that women experience anxiety more than men because they are more sensitive to emotions. Hence, it affects their feelings of anxiety.

Alshammari^(13)^ states that practical learning experiences and theory are significant in nursing education, so students who have never studied nursing theory or practice experience higher anxiety levels. According to Kuraesin in Malfasari^(14)^, educational background is one of the social factors associated with student anxiety, so individuals with different educational backgrounds will have other anxiety behaviors. This aligns with Hutagalung & Siagian’s^(15)^ research, which states a significant relationship between background education and anxiety with an Asymp Sig value of 0.019 <0.05.

Based on the research conducted by Malfasari et al.^(14)^ shows that as many as 53.4% of students regard the clinical practice environment as a positive one. Lindasari, Nuryani & Sopiah^(16)^ stated that a sense of trust given by health workers to students undergoing clinical practice would assist in increasing student self-confidence to minimize worry. Therefore, the author is interested in looking at the variables related to nursing students’ anxiety in carrying out clinical practice.

## OBJECTIVES

To identify the relationship of gender, educational background, and learning environment with student anxiety-related clinical practice.

## METHODS

### Ethical Aspects

Researchers have received a letter of passing the ethical review from the UPH Faculty of Nursing Committee Ethics. Researchers provided research information on an online questionnaire and then asked for willingness to participate, giving informed consent. The researcher ensured the confidentiality of the respondents by not asking for initials or full names (anonymity). Raw data can only be accessed by researchers and used for research. The data that comes out is only data that has been processed.

### Design, period, and place of study

This study used a correlational quantitative descriptive design with the equator instrument STROBE. This research was conducted online using a modified questionnaire instrument: the Zung Self-Rating Anxiety Scale (ZSAS) and Clinical Learning Environment Supervision Nurse Teacher (CLES+T)^(17)^. The questionnaire was distributed via a Google form using a Likert scale. This research was conducted from 30 March to 28 April 2022 in the Faculty of Nursing in Tangerang.

### Population and sample: inclusion and exclusion criteria

The population in this study was 233 2^nd^ year nursing students at one Faculty of Nursing in Tangerang. Using a probability sampling technique with a simple random sampling method, and obtained a sample of 135 students with a response rate of 91.2 %. Inclusion criteria in this study were 233 2nd-year nursing students. Exclusion criteria in this study were cohort students in the 2^nd^ year and students who have become respondents to the validity test and reliability.

### Study protocol

To carry out this study, two instruments were used. Validity and reliability tests were conducted on 32 respondents. The researcher has determined the value of the r table using the product moment table, which is 0.349. Based on the validity test, there were 16 valid and four invalid statements on the modified ZSAS questionnaire, one invalid statement, and 34 valid statements on the CLES+T modified questionnaire. The research team has corrected the weak statement, and the supervisor has checked it. The reliability test was carried out and obtained Cronbach’s alpha value of 0.893 on the modified ZSAS questionnaire and 0.950 on the modified CLES+T questionnaire. The reliability test results questionnaires showed a value of Cronbach’s alpha > 0.7, so both questionnaires were declared reliable. The data obtained were analyzed using the Chi-Square statistical test using SPSS version 28.

Permission from the faculty related to the study allowed the authors to distribute the link questionnaires to all students through an online platform (WhatsApp). One hundred thirty-five students agreed to join the study and gave informed consent, available online. Afterward, each student could access the questionnaire to be filled out and complete all the given questions. Every respondent included in the inclusive research criteria must fill out informed consent in advance and understand that students can recover from research at any time and that there is no coercion.

### Analysis of results and statistics

The study was carried out in univariate and bivariate data analysis. The data were analyzed in the Statistical Package for the Social Sciences (SPSS) version 28 using the Kolmogorov test to test the normality of the data distribution, and all data are normal. Univariate data is presented with a frequency distribution. In bivariate statistics, the data obtained were analyzed using the Chi-Square statistical test using SPSS version 28. The researcher chose Chi-square because the data used is categorical and wants to see the relationship between more than two variables.

## RESULTS

This section presents univariate data such as gender, educational background, learning environment, and nursing students’ anxiety.

Based on Table 1, shows that the respondents in this study were dominated by female students at 90.4%. Respondents in this study showed that 80.7% had a high school educational background majoring in science. The results show that 65.94% of nursing students consider the clinical practice environment to be a negative learning environment. Respondents in this study also showed that 88.9% experienced mild anxiety.

**Table 1:** Characteristics of Nursing Students at One Nursing Faculty in Tangerang April 2022 (n=135)

Category	n	%
Gender		
Female	122	90.4
Male	13	9.6
Educational background		
Natural science major	109	80.7
Social science major	6	4.4
Language major	1	0.7
Nursing major	14	10.4
Majoring in health analysis	2	1.5
Accounting Major	3	2.2
Learning environments Positive environment	46	34.06
Negative environment	89	65.94
Anxiety level		
Normal	3	2.2
Mild	120	88.9
Moderate	12	8.9
Heavy	0	0
Panic	0	0
Total	135	100

This section is about the correlation of all the factors.

Table 2 shows that female students dominate the respondents in this study at 90.4%. The results show that 88.9% of respondents experienced mild anxiety. This table also shows that 80.74% of female students and 8.15% of male students experienced mild anxiety related to clinical practice. Based on the chi-square statistical test, the Asymp value was obtained. The sig likelihood ratio on the gender variable is 0.517 > 0.05, so it can be concluded that there is no significant relationship between the type of gender variable and the anxiety variable.

**Table 2:** Relationship of Gender Factors with Anxiety of Nursing Students at One Nursing Faculty in Tangerang April 2022 (n=135)

Gender	Normal		Mild		Moderate		Total		*Likelihood ratio*
	n	%	n	%	n	%	n	%	
Female	2	1.48	109	80.74	11	8.15	122	90.4	0.517
Male	1	0.74	11	8.15	1	0.74	13	9.6	
Total	3	2.22	120	88.99	12	8.89	135	100	

Table 3 shows that 80.7% have a high school education background in a science major. The results show that 88.9% of respondents experienced mild anxiety. Based on this table, 74.81% of students with a high school education background majoring in natural science and 8.15% of students with a nursing vocational education background experienced mild anxiety related to clinical practice. Based on the chi-square statistical test, the Asymp value was obtained. The sig likelihood ratio on the educational background variable is 0.227 > 0.05, so it can be concluded that there is no significant relationship between educational background variables with the anxiety variable.

**Table 3:** Relationship of Educational Background Factors with Anxiety of Nursing Students at One Nursing Faculty in Tangerang April 2022 (n=135)

Educational background	Normal		Mild		Moderate		Total		*Likelihood ratio*
	n	%	n	%	n	%	n	%	
Natural science major	2	1.48	101	74.81	6	4.44	109	80.7	0.227
Social science major	1	0.74	4	2.97	1	0.74	6	4.4	
Language major	0	0	0	0	1	0.74	1	0.7	
Nursing major	0	0	11	8.15	3	2.23	14	10.4	
Majoring in health analysis	0	0	2	1.5	0	0	2	1.5	
Accounting Major	0	0	2	1.5	1	0.74	3	2.2	
Total	3	2.22	120	88.99	12	8.89	135	100	

Based on Table 4, 65.94% considered the clinical practice a negative learning environment. The results show that 88.9% of respondents experienced mild anxiety. Based on the chi-square statistical test, the Asymp value is obtained. The sig likelihood ratio on the environmental variable is 0.203 > 0.05, so it can be concluded that there is no significant relationship between environmental variables and anxiety variables.

**Table 4:** Relationship of Environmental Factors with Anxiety of Nursing Students at One Nursing Faculty in Tangerang April 2022 (n=135)

Environmental	Normal		Mild		Moderate		Total		*Likelihood ratio*
	n	%	n	%	n	%	n	%	
Positive environment	2	1.48	42	31.1	2	1.48	46	34.06	0.203
Negative environment	1	0.74	78	57.8	10	7.4	89	65.94	
Total	3	2.22	120	88.99	12	8.89	135	100	

## DISCUSSION

### The Relationship of Gender Factors with Nursing Student Anxiety Related to Clinical Practice

Table 2 shows that female and male students experienced mild anxiety related to clinical practice. This result differs from a study by Coelho et al.^(18)^, who reported that female students showed moderate and severe anxiety scales. This looks dominant in women because the number of both genders is indeed unequal. If we look at the proportions of each group of men and women, they still experience almost equal anxiety. According to Rollinson & Kish in Rahmawati, Sukmaningtyas & Muti^(19)^, nursing students are dominated by women because nursing has a history of care-taking roles in families and communities who have diligent, thorough, patient, and painstaking attitudes that women generally possess. Nogueira *et al.* ^(20)^ also found that nursing has historically been a predominantly female profession. This study’s results align with Sari’s^(6)^ on students at the Nursing Academy, which showed that 40.6% of nursing students experienced mild anxiety during clinical practice despite having previous experience in hospitals. Another study by Lindasari, Nuryani & Sopiah^(16)^ on 139 nursing students also stated that 36.04% of nursing students experienced mild anxiety, which was influenced by their preparation before clinical practice at the hospital.

Based on the chi-square statistical test, it can be concluded that there is no significant relationship between the type variable gender and the anxiety variable. This could be because the process of stress is an intrapersonal process that can be faced by anyone in an area that is uncomfortable for them, regardless of gender. This is not in line with research conducted by Iswanti, Suratih & Winasti^(21)^, which states that there is a relationship between student gender and anxiety in participating in clinical practice. The results of the research that has been carried out show that 90.4% of female students experience anxiety related to clinical practice. This is supported by research conducted by Jayanti, Krisnawati & Devi^(12)^ which states that most students who experience anxiety about nursing clinical practice are female. According to a study by Fahrianti & Nurmina^(22)^ female students are known to have more anxiety disorders than boys. Several factors can cause anxiety; for example, women are more sensitive and think about their inability to do something, and men feel more logically and focus on the causes of problems. Another study by Rahmawati, Sukmaningtyas & Muti^(19)^ found that 75.7% of research respondents are female.

### The Relationship of Educational Background Factors with Nursing Students’ Anxiety Regarding Clinical Practices

This study found that nursing students experienced mild anxiety related to clinical practice. Based on Table 3, most of the respondents in this study had a high school educational background majoring in science (80.7%). And 74.81% of students with a high school education background majoring in science experienced mild anxiety related to clinical practice. This is in line with the research of Natalia *et al*.^(21)^ which stated that 91.5% of students in one of the nursing faculties in Tangerang came from high schools in general, namely majoring in science and social studies. The chi-square test results show no significant relationship between the educational background variable and anxiety with the Asymp Sig likelihood ratio. This aligns with research conducted by Vellyana et al.^(10)^, which states no relationship between educational background and anxiety. This study is not in line with Natalia *et al.* ^(23)^ who say that there is a relationship between educational background and student anxiety related to clinical practice because students with a nursing vocational education background learn more about nursing theory and training before clinical practice.

However, this is inversely proportional to the results of existing research because almost all students with a vocational education background majoring in nursing experienced mild anxiety, which means that even though previously students had studied nursing theory and practice at school, they would still experience anxiety when facing clinical practice.

### Relationship of Environmental Factors with Nursing Student Anxiety Related to Clinical Practice

Table 4 shows that the chi-square correlation test results indicate that there is no significant relationship between environmental variables and nursing student anxiety related to clinical practice. This differs from the results of research conducted by Malfasari *et al.^(^* ^14)^ with Asymp. Sig 0.045 <0.05 indicates a significant relationship between the environment and nursing student anxiety related to clinical practice.

Most respondents in this study considered the clinical practice environment unfavorable (65.94%). This is in line with Moraes *et al.* ^(24)^, who state that experience and adaptation to the new environment may occur unsuccessfully, which can cause negative feelings to arise, which can be reflected in personal life and the learning process of students and can lead to suicide risk.

This study is inversely proportional to Malfasari *et al*.’s^(14)^ survey, which showed that 53.4% of nursing students considered the clinical practice environment a positive environment. However, we, as part of the faculty, need to realize as well as Amaral *et al.* ^(25)^ said, that the environment as a significant anxiety factor needs to be modified because of the difficulty of harmonizing personal and academic life (clinical practice experience), which is proven to reduce sleep quality. On the other hand, nursing students must also learn to adapt and carry out clinical practice under the guidance of a clinical supervisor. As Santos et al.^(26)^ suggested, anxiety will be reduced if accompanied by professionals responsible for training, learning, and developing student skills.

### Study limitations

This research used an online questionnaire, which means we cannot explain directly about research information and cannot ask for informed consent directly from respondents. The way to overcome this so that it doesn’t affect the results is in the Google research form, several pages are created, namely the first page explaining the research, and the second page is informed consent. Then if the respondent is willing to be a respondent, it will continue to the research questions page. However, if the respondent is not willing, they will be directed to send a questionnaire without going to the question page. The other limitation is sample group is only one batch, so it cannot see the difference in anxiety and the variables that relate to it; it will be more varied if the sample is taken in several batches. The way to overcome this so that it doesn’t affect the results is by using a sample of more than 100 respondents.

### Contributions to nursing and health

This research is expected to be used as information that gender, educational background, and environment have no significant relationship to students’ anxiety in nursing-related clinical practice. In addition, this research can also be used to add insight, knowledge, and sources of supporting data in researching the other variables or factors of student anxiety. This research is expected to be used as a guideline for clinical practice for nursing students who are undergoing clinical practice to find out the description of their anxiety and suggest that the faculty evaluate students to find out whether there are students who experience anxiety related to clinical practice.

## CONCLUSIONS

The respondents of this study were students of the 2^nd^ year nursing students at a private university nursing faculty in Tangerang. The environmental variables in this study showed that nursing students considered the clinical practice environment negative. No significant relationship exists between gender, educational background, and environmental variables with anxiety. Seeing the results of students’ anxiety levels, students need to be given more preparation before entering the practical field

The results of this study can also become a basis for clinical coordinators of related faculties and units to improve and modify clinical practice environments to be positive for nursing students. The researcher also recommends that future researchers conduct qualitative research on anxiety-related factors.
